# In/opposed phase imaging effectively differentiates fat from enhanced myocardium in patients with myocardial late gadolinium enhancement

**DOI:** 10.1186/1532-429X-11-S1-T1

**Published:** 2009-01-28

**Authors:** Denise Kleindienst, Hassan Abdel-Aty, Wolfgang Utz, Andre Rudolph, Jeanette Schulz-Menger

**Affiliations:** Franz-Volhard-Klinik, Kardiologie, Charité Campus Buch, Universitätsmedizin Berlin, Helios-Klinikum, Berlin Germany

**Keywords:** Late Gadolinium Enhancement, Late Enhancement, Additional Diagnostic Information, Late Enhancement Imaging, Late Gadolinium Enhancement Area

## Background

Suppressing fat in late gadolinium enhancement (LGE) images is a challenging task in a variety of clinical settings. Both fat and LGE appear bright in these images making it difficult to quantify the exact extent of LGE or even in some cases e.g. in myocarditis to establish the presence of late enhancement, which because of its subepicardial location may be easily confused with pericardial fat. We tested the clinical utility of a novel in/opposed phase LGE sequence in this setting.

## Methods

We implemented a cardiac gated two-dimensional inversion recovery prepared pulse sequence to acquire gradient echo images (in and opp.-LGE) with two echo times (2.4 and 5.0 ms) 10 minutes after the IV injection of gadolinium DTPA (0.2 mmol/Kg BW) in a single breath hold. This was achieved at the cost of an increase in bandwidth (140 vs. 400 Hz/Px) in comparison to the standard LGE sequence. All other sequence parameters including the inversion time were kept constant (flip angle 30, matrix 148 × 256, slice thickness/gap: 7/3 mm) Images were acquired twice once using the conventional non-fat sat LGE (con-LGE) sequence and then using the proposed in/opposed phase approach in 28 patients with positive LGE secondary to ischemic and non-ischemic conditions. Images were evaluated both quantitatively and qualitatively. Contrast to noise (CNR) ratios between fat, normal and enhanced myocardium were measured. One independent observer visually evaluated the diagnostic quality of all images.

## Results

**Figure 1:**
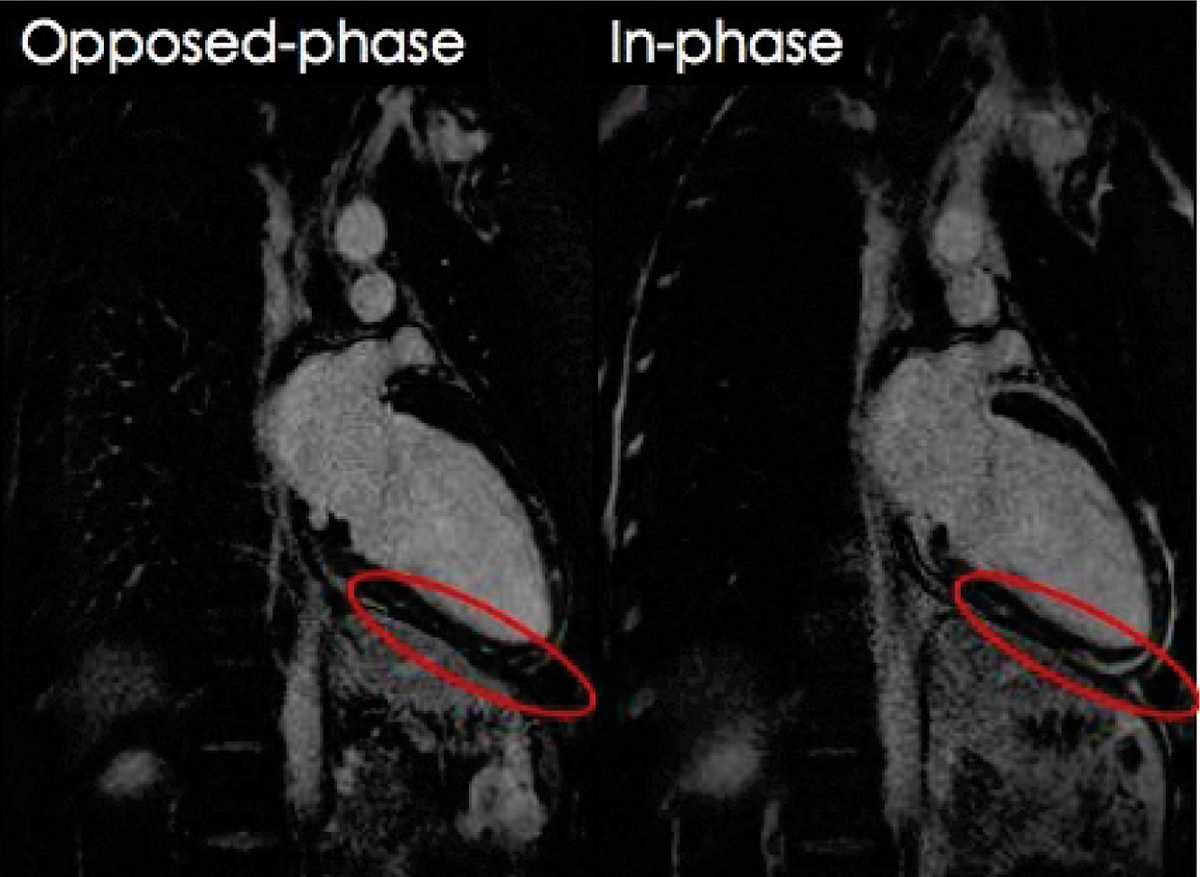
Figure 1

Fat was homogenously suppressed over the entire FOV in all cases. The CNR between fat and normal myocardium decreased significantly in opp-LGE compared to con-LGE (49.5 ± 20 vs. 10.6 ± 8; p < 0.0001) indicating effective fat suppression. Furthermore, the CNR between LGE areas and the pericardial fat increased significantly in opp-LGE compared to con-LGE (10.9 ± 12 vs. -9.9 ± 22; p < 0.0001) implying better LGE-fat differentiation. The qualitative assessment proved the sequence robustness over a wide range of indications and that this approach could provide additional diagnostic information over that obtained from con-LGE in 2/28 patients. Figure 1.

## Conclusion

We provide a first clinical utilization of a novel fat suppression late enhancement imaging combining in/opposed phase imaging with conventional inversion recovery gradient echo sequences. This approach enables effective differentiation between fat and enhanced myocardium, which should result in accurate LGE quantification.

